# The predictive power of Bitcoin prices for the realized volatility of US stock sector returns

**DOI:** 10.1186/s40854-023-00464-8

**Published:** 2023-03-06

**Authors:** Elie Bouri, Afees A. Salisu, Rangan Gupta

**Affiliations:** 1 School of Business, Lebanese American University, Byblos, Lebanon; 2 Centre for Econometrics and Applied Research, Ibadan, Nigeria; 3 Department of Economics, University of Pretoria, Private Bag X20, Hatfield, 0028 South Africa

**Keywords:** Bitcoin prices, S&P 500 index, US sectoral indices, Realized volatility prediction, Economic gains

## Abstract

This paper is motivated by Bitcoin’s rapid ascension into mainstream finance and recent evidence of a strong relationship between Bitcoin and US stock markets. It is also motivated by a lack of empirical studies on whether Bitcoin prices contain useful information for the volatility of US stock returns, particularly at the sectoral level of data. We specifically assess Bitcoin prices’ ability to predict the volatility of US composite and sectoral stock indices using both in-sample and out-of-sample analyses over multiple forecast horizons, based on daily data from November 22, 2017, to December, 30, 2021. The findings show that Bitcoin prices have significant predictive power for US stock volatility, with an inverse relationship between Bitcoin prices and stock sector volatility. Regardless of the stock sectors or number of forecast horizons, the model that includes Bitcoin prices consistently outperforms the benchmark historical average model. These findings are independent of the volatility measure used. Using Bitcoin prices as a predictor yields higher economic gains. These findings emphasize the importance and utility of tracking Bitcoin prices when forecasting the volatility of US stock sectors, which is important for practitioners and policymakers.

## Introduction

Previous studies tend to consider the relationship between Bitcoin prices and the aggregate US stock market index (see, among others, Bouri et al. 2017a; Baur et al. 2018; Das et al. 2020; Naeem et al. 2020; Shahzad et al. 2020; Koutmos et al. 2021; Bouri et al. 2022),^1^ pointing to potential diversification benefits arising from the detachment of Bitcoin from the global financial system. Notably, studies examining the Bitcoin–stock nexus at the sectoral level of US stock data are few and limited. For example, Symitsi and Chalvatzis (2018) and Bouri et al. (2020a) focus on technology stocks and find evidence of significant linkages. According to Xu et al. (2022), the returns of blockchain and crypto-exposed US companies and major cryptocurrencies all jump together. However, the US stock market includes heterogenous stocks from not only the information technology sector, but also from ten different sector indices covering financials, telecommunications, and energy and gas, to name a few. Many US investors use sector rotation strategies in response to changes in market conditions and economic cycles.^2^ Although preliminary, the consensus from those studies suggests that the magnitude and sign of the relationship between Bitcoin and US stock returns are sector dependent (see, Bouri et al. 2020a, b), indicating the utility of conducting an analysis at the sectoral level of US stock data.

Stock volatility is important to traders, investors, and policymakers in addition to stock returns. Indeed, given the importance of exogenous variables in forecasting models, option pricing, volatility trading strategies, and portfolio allocation and risk management,^3^ many practitioners are keen to understand more about them. Therefore, it would be useful to provide comprehensive evidence on whether Bitcoin prices contain valuable information that can be used to forecast stock volatility at the aggregate and sectoral levels. However, there is no evidence that Bitcoin prices can predict the volatility of the US stock market index and its various sector indices. Addressing this question is timely and relevant given that many retail and institutional stock investors in the United States view Bitcoin as an investment or trading venue. Furthermore, policymakers are looking for ways to leverage the cryptocurrency universe to launch a digital US dollar or a central bank digital currency.

From policy and investment perspective, numerous compelling reasons exist to examine the Bitcoin–US stock nexus. First, policymakers are concerned about sustaining economic growth and therefore are constantly under immense pressure to understand macroeconomic variables capable of predicting stock market behavior, thereby influencing policy implementation. Therefore, understanding the predictive power of Bitcoin prices for the US stock volatility will aid relevant policymakers in developing policies to sustain economic growth. Second, stock market performance is regarded as an important indicator of macroeconomic stability within an economy, as well as a means of attracting foreign investment. Thus, policymakers must ensure stock market stability by monitoring risk factors that could distort its stability.^4^ Third, in the valuation of investment securities, investors are frequently interested in information on market risk and its relevant predictors, which helps minimize risks and improve risk-adjusted returns. In sum, as long as the US stock market is an integral part of the US economy, discussions and analyses about risk predictors will be prominent among investors and policymakers. Motivated by evidence of stronger ties between Bitcoin and the US stock markets in the last 2 years (Kristoufek 2020; Kumar et al. 2022) and a lack of empirical evidence on the predictive power of Bitcoin prices for the volatility of US stock indices, particularly at the sectoral level, the goal of this paper is to examine Bitcoin prices’ ability to predict the realized volatility of the S&P 500 composite index and its 11 sector indices. Methodologically, we apply Westerlund and Narayan’s (2012, 2015) model, which accounts for key salient data features, such as endogeneity, persistence, and conditional heteroscedasticity. We conduct in-sample and out-of-sample analyses over multiple forecast horizons. Our analysis allows for possible structural breaks within the model framework to enhance the predictive capability of the applied model (Salisu et al. 2019a). Furthermore, we chose realized volatility because of its inherent ability to provide an observable and unconditional metric of volatility (model-free), whereas other volatility models, such as conditional volatility models and stochastic volatility models,^5^ would have been a latent process (see Chan and Grant 2016; Bouri et al. 2021). Still, we consider conditional volatility obtained from GARCH modeling to ensure the robustness of our main results.

Our main findings show that Bitcoin prices have an inverse relationship with the realized volatility of US stock indices. Regardless of stock sector or forecast horizon, the predictive model that accounts for Bitcoin prices and salient features of data outperforms the benchmark historical average model. Notably, incorporating Bitcoin prices as a predictor results in higher economic gains across a wide range of US stock sector indices. These results indicate the importance and utility of closely monitoring Bitcoin prices when forecasting the realized volatility of US stock sectors.

Our methodology and findings contribute to three lines of investigation. The first line is about the literature on Bitcoin–US stock market links, which appears to show mixed findings. For example, some previous studies show a weak relationship and thus hedging and safe haven implications for asset allocation and risk management (Bouri et al. 2017a; Baur et al. 2018; Shahzad et al. 2020), whereas others find a stronger relationship after the pandemic jeopardizes Bitcoin’s hedging ability (see, Conlon and McGee 2020; Kristoufek 2020; Kumar et al. 2022). Elsayed et al. (2022), Jiang et al. (2022), Maghyereh and Abdoh (2022), and Akyildirim et al. (2021) have all documented significant volatility links between aggregate stock market returns and the Bitcoin market. Furthermore, the majority of previous studies have attempted to explain Bitcoin volatility (see, e.g., Walther et al. 2019; D’Amato et al. 2022; Sapkota 2022; Wang et al. 2022) or focused on the predictability of major cryptocurrencies and the profitability of trading strategies using machine learning techniques (Sebastião and Godinho 2021),^6^ whereas our paper has a different scope by focusing on Bitcoin price ability to predict stock volatility. The second line of investigation focuses on Bitcoin and sectoral stock indices. For example, at the sectoral level of stock data, Bitcoin and US sector returns appear detached or weakly related, which has significant hedging implications (Bouri et al. 2020a). Given the need for specialized and powerful computers for Bitcoin mining, information technology firms that manufacture computer hardware and software should be involved in the Bitcoin market. In fact, academic research focuses on specific sectors, such as technology (Bouri et al. 2020b) or technology and energy stocks (see, Symitsi and Chalvatzis). For example, Symitsi and Chalvatzis (2018) used generalized autoregressive conditional heteroscedasticity (GARCH)-based models to demonstrate significant return and volatility links between Bitcoin and energy and technology companies. Rathi (2022) provides evidence of the cryptocurrency market’s impact on the semiconductor industry. Recent press articles highlight Bitcoin’s ability to predict the dynamics of US stock market indexes, particularly technology stocks. They argue that “investors are fleeing riskier assets from tech stocks to cryptocurrencies as the Federal Reserve weighs whether to launch a US digital currency.”^7^ The third line of investigation is concerned with the relationship between Bitcoin and US stock indices, which can be subject to structural breaks due to upsetting and irregular events (see, Ciaian et al. 2016; Salisu et al. 2019c). As a result, we account for structural breaks in the predictive models for volatility of US sector indices, and we then assess both the in-sample and out-of-sample predictive contents of Bitcoin prices, as well as other salient features of the series. Finally, we provide some utility metrics for assessing the value of observing Bitcoin prices when valuing stock market risk.

The remainder of our paper is organized as follows. "Related studies" section examines the literature on the relationship between Bitcoin and traditional markets, particularly stock market indices. "Methodology" section describes the methodology. "Data Description" section contains the dataset. "Results" section presents and discusses the results, and conducts a robustness analysis. "Conclusion" section concludes with a discussion of policy implications.

## Related studies

Bitcoin has emerged in 2009 as a decentralized cryptocurrency independent of the global financial system, propelled by distinct factors centered on its innovative blockchain technology and attractiveness.^8^ Bitcoin’s hedging ability, particularly for the general stock market, is well known (see, Bouri et al. 2017a, 2020b; Baur et al. 2018; Das et al. 2020; Naeem et al. 2020; Shahzad et al. 2020; Koutmos et al. 2021). However, recent Bitcoin market dynamics in the post-pandemic period show a less detached and more synchronized market with the US stock market, as represented by the S&P 500.^9^ As indicated by the International Monetary Fund, the closer connections between Bitcoin and US stocks raise “the risk of contagion across financial markets—the correlation coefficient of their daily moves was just 0.01—but that measure jumped to 0.36 for 2020–2021 as the assets moved more in lockstep, rising together or falling together.” Kumar et al. (2022) pointed to a stronger relationship between Bitcoin and US stock markets as a result of its progress toward mainstream finance, the investment community, and exchange traded funds. Other studies conducted during the pandemic period yielded similar results. For example, Kristoufek (2020) questions Bitcoin’s role as a safe haven. Conlon and McGee (2020) argue that “the S&P 500 and Bitcoin move in lockstep, resulting in increased downside risk for an investor with an allocation to Bitcoin.”^10^

Recent studies have revealed a certain level of connectedness, in some cases low, between Bitcoin and other important assets, particularly during the non-crisis period, though shocks are thought to influence markets with dissimilar macroeconomic factors, leading to heightened cross-market interconnectedness (Kumar et al. 2022). In essence, this means that if Bitcoin’s price falls suddenly, it may affect investor sentiment and, as a result, other markets such as the stock market (see Attarzadeh and Balcilar 2022). Given the level of market integration in advanced economies like the United States, the possibility of such a spillover effect is even more plausible. However, for the purpose of our study, we do not assume that this occurs uniformly across all stock sectors. In fact, given previous evidence that stock sectors tend to respond differently to macroeconomic fundamentals, disaggregation becomes even more important to better appreciate potential heterogeneity in the relationship between the variables. For example, during the COVID-19 pandemic, the value of healthcare stocks increased, whereas stocks in the energy, industrials, materials, real estate, travel, and tourism sectors declined.^11^ Furthermore, the underlying technology of Bitcoin, blockchain,^12^ has been adopted in many applications in information technology and Fintech companies. Moreover, many computer hardware firms produce chips to power Bitcoin mining, supporting the academic literature’s argument that the relationship between Bitcoin and US stocks can be sector dependent. In this regard, we should not overlook cryptocurrency mining, which is an energy-intensive activity that results in a close relationship between cryptocurrency and energy markets (Ji et al. 2019). Bouri et al. (2020b) examine Bitcoin’s ability to hedge against the downside risk of the US stock sectors and find evidence of sector heterogeneity. In a separate study, Bouri et al. (2020b) use a Granger causality approach to examine the predictability of Bitcoin and US technology stock returns and report some significant causal flows. Our paper differs in its focus, which is the ability of Bitcoin prices to predict volatility in US stock sector indices, and the method used, which is Westerlund and Narayan (2012, 2015). The latter offers several advantages, which are detailed below.

## Methodology

As mentioned in the introduction section, our model assumes that the Bitcoin market can be a major risk factor for the US stock market, and thus Bitcoin prices should affect the volatility of US stock sectors. However, the effect may differ across US stock sectors depending on the degree of proximity between each sector’s activities and business models and the Bitcoin market and the applications of its underlying blockchain technology.

In terms of estimation, we use a Westerlund and Narayan (2012, 2015) [WN]-Type distributed lag model to examine the relationship between realized volatility of US sector stocks and Bitcoin prices. Endogeneity, persistence, and conditional heteroscedasticity are all important data features of financial series that the WN-Type predictive model simultaneously accounts for. In addition to Westerlund and Narayan’s (2012, 2015) theoretical results validating the inclusion of these features in predictability analyses, several empirical studies using this methodology have reported the same outcome (see, Narayan and Bannigidadmath 2015; Narayan and Gupta 2015; Phan et al. 2015; Bannigidadmath and Narayan 2016; Devpura et al. 2018; Salisu et al. 2019a, b, c, d, 2021; among others)). To improve the applied model’s predictive capability, we account for possible structural breaks within the model framework by including break dummies obtained using the Bai and Perron (2003) test, which allows for up to five breaks. Accounting for significant structural breaks improves outcome predictability (see Salisu et al. 2019a, b, c; among others).

We therefore define our predictive model as follows^13^:

1 $$RV_{t} = \alpha + \beta btc_{t - 1} + \gamma \left( {btc_{t} - \rho btc_{t - 1} } \right) + \sum\limits_{i = 1}^{5} {\delta_{i} brk_{it} } + \varepsilon_{t}$$

where $RV_{t}$ is a 20-day annualized realized volatility from the corresponding US sector stock returns computed at period $t$; $btc_{t}$ is the log-transformed Bitcoin price at time $t$; $brk_{it}$ is the $ith$ break dummy; $\alpha$ is the intercept; $\beta$ is the coefficient associated with our predictor variable of interest, which gives the stance of predictability, or otherwise; the term $\left[ {\gamma \left( {btc_{t} - \rho btc_{t - 1} } \right)} \right]$ is a persistence-adjustment term introduced to resolve the inherent persistence effect and endogeneity bias caused by model mis-specification; $\delta_{i}$ is the coefficient associated with the break dummy; and $\varepsilon_{t}$ is a zero mean idiosyncratic error term. The break dates are determined using the Bai and Perron (2003) multiple breakpoint test, which involves regressing each US stock return’s realized volatility on a one period lag of the log-transformed Bitcoin price series, with a maximum of five breaks allowed. The underlying predictability test has the null hypothesis $\left[ {H_{0} :\beta = 0} \right]$ against a mutually exclusive alternative, $\left[ {H_{a} :\beta \ne 0} \right]$, where a rejection (non-rejection) of the null hypothesis implies the predictability (no predictability) of Bitcoin price for realized volatility of US sector stock. Given that our data are collected on a daily (high) frequency and the possibility of conditional heteroscedasticity, we weight Eq. (1) with the standard deviation of the residuals obtained from a $GARCH\left( {1,1} \right)$ model to account for possible conditional heteroscedasticity effect. We also estimate the resulting equation with ordinary least squares to obtain the feasible quasi generalized least squares estimates.

The out-of-sample forecast evaluation of our WN-Type predictive model relative to a benchmark historical average model that does not take into account the predictive information inherent in the Bitcoin price series is a follow-up to the predictability stance. As a result, we subjected our predictive model’s forecast to statistical evaluations using the traditional root mean square error Clark and West (CW) (2007), and economic significance. Based on existing research (see Narayan and Gupta 2015; among others) that have shown the insensitivity of estimation outcomes to the choice of data split, the 75:25 data split option is considered for in-sample estimation or predictability and out-of-sample forecast evaluation, respectively. On the out-of-sample period, we consider forecast horizons of 30, 60, and 120 days ahead using a rolling window framework that allows for some time variation.

We can formally determine the statistical significance of the observed difference between the forecast errors of the contending models using the CW test, which compares the predictive accuracy of two competing models. The CW framework is as follows:

2 $$\hat{f}_{t + k} = \left( {r_{t + k} - \hat{r}_{1t,t + k} } \right)^{2} - \left[ {\left( {r_{t + k} - \hat{r}_{2t,t + k} } \right)^{2} - \left( {\hat{r}_{1t,t + k} - \hat{r}_{2t,t + k} } \right)^{2} } \right]$$

where $k$ denotes the forecast period, $\left( {r_{t + k} - \hat{r}_{1t,t + k} } \right)^{2}$ and $\left( {r_{t + k} - \hat{r}_{2t,t + k} } \right)^{2}$ are respectively the squared errors for the restricted and the unrestricted models, and $\left( {\hat{r}_{1t,t + k} - \hat{r}_{2t,t + k} } \right)^{2}$ is the adjusted squared errors introduced by the CW test to correct any noise associated with the larger model’s forecast. Thus, the sample average of $\hat{f}_{t + k}$ can be expressed as Mean Squared Error (MSE with $MSE_{1} - \left( {MSE_{2} - {\text{adj}}{.}} \right)$, and each term is computed as $MSE_{1} = P^{ - 1} \sum {\left( {r_{t + k} - \hat{r}_{1t,t + k} } \right)^{2} }$,$MSE_{2} = P^{ - 1} \sum {\left( {r_{t + k} - \hat{r}_{2t,t + k} } \right)^{2} }$, and ${\text{adj}}. = P^{ - 1} \sum {\left( {\hat{r}_{1t,t + k} - \hat{r}_{2t,t + k} } \right)^{2} }$, where $P$ denotes the number of predictions used in computing these averages. The equality of forecast performances between the restricted and unrestricted models is tested by regressing the $\hat{f}_{t + k}$ on a constant and drawing inference based on the resulting t-statistic of the constant. Given the null hypothesis of equality of mean squared errors, the rejection criteria are whether the resulting t-statistics is greater than + 1.282 (for a one-sided 0.10 test) or + 1.645 (for a one-sided 0.05 test).

## Data description

The data set includes daily Bitcoin prices and 20-day annualized realized volatility of the US S&P 500 composite index returns and its 11 sector stock returns (Consumer Discretionary, Consumer Staples, Energy, Financials, Health Care, Industrials, Information Technology, Materials, Real Estate, Telecommunication Services, and Utilities). The motivation for using stock sector indices in addition to the aggregate composite index stems from the diverse reaction of US stock sectors to exogenous variables and risk factors (Shahzad et al. 2021a). Indeed, some sectors are highly dependent on technological revolution and innovation, and thus may be related to Bitcoin and its blockchain technology (Bouri et al. 2020a, b). In contrast, others are more related to the economy’s traditional industrial and manufacturing segments and thus less related to the Bitcoin market. The log returns of the S&P 500 composite index and each of its 11 sector stock indices are calculated as the difference in logarithmic prices between two consecutive daily prices. Then, they are used to compute realized volatility based on the sum of squared returns using 20 trading days in a month as the rolling window size, which is then annualized using 252 trading days in a year.^14^ All data were collected from DataStream between November 22, 2017, and December 30, 2021. According to Bitstamp, one of the oldest and most established Bitcoin exchanges, Bitcoin prices are relative to the US dollar.

Table 1 contains a detailed summary of the data characteristics in terms of location, spread, and shape, as well as preliminary results on conditional heteroscedasticity, first and higher-order autocorrelation, and persistence effects. The log-transformed Bitcoin price series averages 9.43 with a standard deviation of 0.83 and is found to be positively skewed and platykurtic over the time studied (exhibiting kurtosis value below that of the normal distribution). The result of the realized volatility of the US sector stock returns shows that energy stocks have the highest mean realized volatility and the highest deviation value, implying that energy is the most risky sector. Following that are Information Technology and Financials. Although the price of some health-related stocks increased during the pandemic period, the mean and deviation values for healthcare are relatively low. A similar picture can be drawn for Consumer staples. All realized volatilities are positively skewed and have heavier tails than normal (leptokurtic feature), indicating the non-normality of the realized volatility. We find evidence of the ARCH effect (except for Energy), first and higher-order autocorrelation effects, and persistence effects at all specified lags. The foregoing suggests that the optimal model for assessing the relationship between realized volatility and Bitcoin prices is one that adequately accounts for the majority of the observed data features. Our WN-Type predictive model framework is ideal.

**Table 1:** Summary statistics and preliminary analysis

	Bitcoin	Composite	Consumer discretionary	Consumer staples	Energy	Financials	Health Care	Industrials	Info. Technology	Materials	Real Estate	Telecom. Services	Utilities
*Summary statistics*													
Mean	9.43	0.71	0.83	0.61	1.32	0.96	0.71	0.87	0.98	0.89	0.81	0.86	0.76
Deviation	0.83	0.59	0.54	0.47	0.92	0.74	0.5	0.63	0.65	0.57	0.67	0.49	0.65
Skewness	0.65	3.9	3.24	4.52	3.26	3.87	3.83	3.83	3.5	3.85	4.37	3.51	4.69
Kurtosis	2.24	21.66	17.27	26.65	16.4	20.9	20.73	20.76	19.09	20.75	24.32	19.47	26.84
*Conditional heteroscedasticity effects*													
$ARCH\left( 1 \right)$	13.88***	45.89***	3.33*	143.91***	0.05	51.71***	15.44***	8.84***	88.24***	2.58	6.05**	71.64***	99.52***
$ARCH\left( 5 \right)$	29.08***	25.21***	26.72***	43.06***	1.67	23.08***	10.13***	25.61***	21.96***	13.31***	5.60***	21.73***	63.52***
$ARCH\left( {10} \right)$	19.99***	13.99***	15.91***	29.09***	1.53	11.98***	7.58***	16.36***	11.85***	8.25***	2.96***	11.10***	40.67***
*First and higher order autocorrelation*													
$Q\left( 1 \right)$	2.15	85.92***	24.35***	109.94***	7.59***	82.48***	49.02***	50.95***	87.94***	49.63***	71.94***	50.73***	94.94***
$Q\left( 5 \right)$	28.01***	385.58***	222.12***	478.94***	96.922***	312.26***	225.05***	274.15***	266.14***	195.50***	319.54***	183.19***	518.90***
$Q\left( {10} \right)$	47.00***	566.07***	324.58***	682.49***	170.96***	456.86***	381.44***	445.09***	379.3***	306.76***	441.44***	243.13***	791.43***
$Q^{2} \left( 1 \right)$	13.77***	44.25***	3.34*	127.53***	0.05	49.59***	15.31***	8.82***	81.96***	2.59	6.05**	67.51***	91.54***
$Q^{2} \left( 5 \right)$	204.07***	156.55***	120.86***	298.51***	8.74	172.29***	58.78***	164.59***	127.29***	80.12***	31.20***	131.32***	408.02***
$Q^{2} \left( {10} \right)$	419.33***	196.16***	139.09***	430.91***	16.81*	204.49***	106.18***	252.16***	147.59***	108.23***	35.41***	139.23***	563.21***
Persistence	0.999***	0.994***	0.992***	0.994***	0.992***	0.994***	0.993***	0.994***	0.993***	0.993***	0.994***	0.991***	0.995***
*Significant break dates* (Bai and Perron 2003)													
Break_1	–	02/01/2019	02/01/2019	07/05/2018	01/23/2019	01/31/2019	02/01/2019	02/01/2019	02/01/2019	02/01/2019	01/23/2019	07/11/2018	02/11/2019
Break_2	–	02/27/2020	02/27/2020	02/21/2019	03/06/2020	03/02/2020	02/25/2020	03/03/2020	02/25/2020	02/27/2020	02/28/2020	02/28/2019	03/02/2020
Break_3	–	10/08/2020	10/08/2020	02/27/2020	10/21/2020	10/12/2020	10/08/2020	10/13/2020	10/08/2020	10/08/2020	10/23/2020	03/02/2020	10/12/2020
Break_4	–	05/21/2021	05/21/2021	10/08/2020	–	–	05/21/2021	–	05/21/2021	05/20/2021	–	10/12/2020	–

The summaries are done for 1,072 observation points, where ARCH(#), Q(#) and Q^2^(#) represent the tests for presence of conditional heteroscedasticity, first and higher-order serial correlations, respectively; and statistical significance implying that the tested feature is present in the series, up to the specified lag #. The ***, ** and * denote statistical significance at 1%, 5% and 10% levels, respectively. The break dates are determined using the Bai and Perron (2003) multiple break point test allowing for a maximum of five lags in a regression of each sector stock returns’ volatility on a one period lag of log-transformed Bitcoin prices

**Fig. 1:**
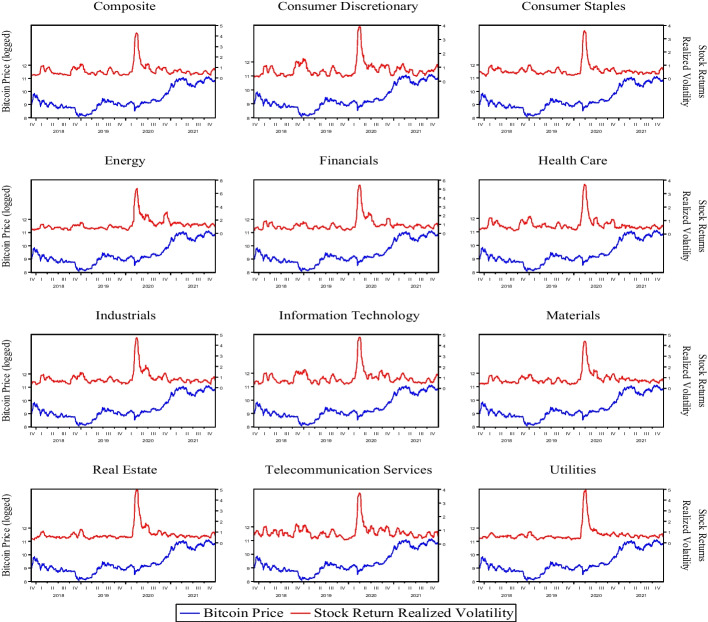
Co-movement between the realized volatility of US stocks and Bitcoin prices. *Note*: The realized volatility is based on the sum of squared returns using 20 days rolling window and subsequently annualized using 252 trading days in a year

Figure 1 depicts the co-movement of US stock returns’ realized volatilities and Bitcoin prices. The paired series exhibit a negative relationship, with observable peaks in realized volatilities of stock returns being matched with troughs in Bitcoin prices, as shown in the figure. We also observe a significant increase and the highest peak in realized volatilities of stock returns on March 27, 2020, which coincides with the period following the declaration of COVID-19 as a pandemic. This suggests evidence of a structural shift in the co-movement of the paired series; however, we use the Bai and Perron (2003) test to test for its presence more formally (see Table 1). We see at least three significant break dates across sector stock realized volatilities, with one of them falling between February 27 and March 3, 2020.

## Results

### Predictability and forecast evaluation

We present the results of our proposed predictive model’s predictability (see Panel A of Table 2) and forecast evaluation (see Panels B and C of Table 2) compared with the benchmark model. Given our interest in demonstrating its predictive value for the realized volatility of US stocks, we only report the estimated coefficient associated with Bitcoin prices. Because the other model components are used to adjust for the observed salient data features, their interpretation would be redundant. The full data sample is used for predictability analyses, whereas we use a 75:25 data split for forecast evaluation, with out-of-sample forecast horizons drawn from the remaining 25% of the full data after using the first 75% to estimate the parameters.

**Table 2:** Predictability and Forecast Evaluation

Sectors	Panel A	Panel B Relative RMSE				Panel C Clark and West (2007)			
	Parameter estimate								
		In sample	Out-of-Sample			In sample	Out-of-sample		
			$\varvec{h} = {\mathbf{30}}$	$\varvec{h} = {\mathbf{60}}$	$\varvec{h} = {\mathbf{120}}$		$\varvec{h} = {\mathbf{30}}$	$\varvec{h} = {\mathbf{60}}$	$\varvec{h} = {\mathbf{120}}$
Composite	− 0.2168*** [0.0037]	0.9030	0.9026	0.9029	0.9009	0.1944*** [0.0123]	0.1885*** [0.0119]	0.1823*** [0.0115]	0.1739*** [0.0108]
Consumer discretionary	− 0.2021*** [0.0004]	0.8571	0.8566	0.8668	0.8774	0.1974*** [0.0161]	0.1949*** [0.0155]	0.1823*** [0.0152]	0.1650*** [0.0101]
Consumer staples	− 0.3673*** [0.0040]	0.9289	0.9267	0.9261	0.9240	0.0611*** [0.0041]	0.0608*** [0.0040]	0.0598*** [0.0038]	0.0588*** [0.0036]
Energy	− 0.6597*** [0.0033]	0.6856	0.7139	0.7262	0.7691	1.2653*** [0.0937]	1.2321*** [0.0906]	1.2057*** [0.0876]	1.1267*** [0.0825]
Financials	− 0.3433*** [0.0029]	0.7650	0.7662	0.7725	0.7744	0.5095*** [0.0471]	0.4947*** [0.0455]	0.4791*** [0.0440]	0.4640*** [0.0412]
Health Care	− 0.3106*** [0.0036]	0.9066	0.9031	0.9035	0.8970	0.1616*** [0.0174]	0.1628*** [0.0168]	0.0890*** [0.0054]	0.0962*** [0.0052]
Industrials	− 0.3990*** [0.0041]	0.7695	0.7686	0.7715	0.7716	0.3466*** [0.0331]	0.3381*** [0.0320]	0.3306*** [0.0309]	0.3239*** [0.0289]
Info. Technology	− 0.3100*** [0.0048]	0.9065	0.9048	0.9074	0.9114	0.2335*** [0.0148]	0.2282*** [0.0143]	0.2207*** [0.0139]	0.2084*** [0.0131]
Materials	− 0.2377*** [0.0021]	0.8448	0.8452	0.8460	0.8453	0.2471*** [0.0161]	0.2389*** [0.0156]	0.2315*** [0.0151]	0.2203*** [0.0142]
Real Estate	− 0.0852*** [0.0007]	0.8415	0.8434	0.8432	0.8452	0.2635*** [0.0197]	0.2555*** [0.0190]	0.2542*** [0.0183]	0.2501*** [0.0172]
Telecommunications	− 0.2381*** [0.0011]	0.8587	0.8580	0.8672	0.8701	0.1254*** [0.0099]	0.1235*** [0.0096]	0.1189*** [0.0093]	0.1179*** [0.0087]
Utilities	− 0.1155*** [0.0005]	0.9609	0.9608	0.9608	0.9599	0.0980*** [0.0037]	0.0946*** [0.0036]	0.0916*** [0.0036]	0.0868*** [0.0034]

The results presented on the table are from the estimation of the WN-Type distributed lag predictive model for Bitcoin prices using realized volatilities of US sectoral stock returns singly as predictors, while simultaneously accounting for inherent persistence, endogeneity, conditional heteroscedasticity and structural breaks. The table comprises three panels: PANEL A presents the in-sample predictability of the realized volatility of US sectoral stock returns for log-transformed Bitcoin price; PANEL B presents the relative root mean square error that compares our WN-Type distributed lag model with the historical average model; while PANEL C presents the Clark and West (2007) test statistics that entails a pairwise comparison of our predictive model with the benchmark historical average model. Under Panels A and C, each cell contain the estimates and the corresponding standard errors in square brackets; while the *** indicates statistical significance at 1% level. Under the PANEL B, values less than unity indicate preference of our predictive model over the benchmark Historical average model; while under PANEL C, our predictive model is adjudged the preferred when the CW statistic is positive and statistically significant

Based on in-sample predictability, we find an inverse relationship between the realized volatility of each of the US sectors and Bitcoin prices, given that the estimated parameter is significantly negative across the stocks in the considered US sectors. This formally validates the observation on the graphical representation of their co-movement, where there are discernible stances of peak-trough matches between realized volatility in US sector stock returns and Bitcoin prices. Implicitly, the level of uncertainty/risk associated with each of the US sector stocks decreases as Bitcoin prices rise and rises as Bitcoin prices fall. This link can be viewed through a risk-return trade-off, where higher returns are associated with higher risks (see French et al. 1987; Bali and Peng 2006; Chiang and Zhang 2018; among others). Our predictability results suggest that higher Bitcoin prices will increase trading (and volatility), implying lower stock trading and volatility. This is especially important in light of studies showing that the Bitcoin market has an asymmetric volatility effect, where positive shocks have a greater impact on volatility than negative shocks of the same magnitude (see Bouri et al. 2017a; Cheikh et al. 2020), and evidence that this asymmetry is not always inverse but depends on scales and market conditions (Kakinaka and Umeno 2021). Lower Bitcoin prices tend to stimulate investment in traditional stocks more than higher Bitcoin prices. The improved stock trading resulting from lower Bitcoin prices will increase stock volatility more than it will decrease when Bitcoin prices fall. Therefore, a hedging relationship somewhat exists between the two assets, which we explore further in a later section titled “Economic Significance,” where we provide potential utility gains derivable by a profit-maximizing investor in the stock market from observing Bitcoin prices. This result supports previous evidence from Dyhrberg (2016) and Bouri et al. (2017a, 2017b); Chan et al. (2019); and López-Cabarcos et al. (2021) indicating Bitcoin’s hedging and safe haven properties for stocks. Overall, Bitcoin prices can signal stock market volatility; additionally, during periods of high stock market volatility, Bitcoin can be used as an effective hedge; thus, policymakers may consider removing institutional barriers currently in place in Bitcoin trading and paying more attention to the dynamics of the Bitcoin market.

The out-of-sample forecast evaluation using the relative root mean square error is shown in Panel B of Table 2. The relative root mean square error is the ratio of our WN-Type predictive model to the benchmark historical average model, with a value less than unity indicating that our predictive model’s forecast errors are less than the benchmark model’s. For ease of interpretation and comparison, the relative root mean square error (RMSE) is adopted here. Based on the presented results, we find that our predictive model for the realized volatility of each US sector stock produces more precise forecasts than the benchmark historical average model, both in-sample and across the specified out-of-sample forecast horizons, because the observed relative RMSE is less than one.

In the same vein, the CW test is a more formal pairwise comparison tool. The estimated statistics must be positive and significant for our predictive model to be chosen as the preferred model when compared to the benchmark historical average model. CW (2007) results show a statistically significant outperformance of our predictive model that accounts for the salient data features, such as endogeneity, persistence, conditional heteroscedasticity, and structural breaks, over the benchmark historical average model that ignores these features in Panel C of Table 2. We find significantly positive coefficients across the specified time periods, indicating that these outperformances persist regardless of the sample period or forecast horizon.

### Economic significance

We conduct an economic-based forecast performance evaluation tool in addition to the statistical-based forecast performance evaluation tool, drawing on Liu et al. (2019) study and Salisu et al. (2020). The economic-based metric is used to determine whether or not incorporating Bitcoin prices as a predictor in our WN-Type distributed lag model provides additional information that yields economic gains over the benchmark historical average model that ignores Bitcoin prices. It is not surprising for a typical mean–variance utility investor to optimize available portfolio among assets and/or investment options, in contrast to a risk free asset. The optimal weight, $w_{t}$, is defined as

3 $$w_{t} = \frac{1}{\gamma }\frac{{\theta \hat{r}_{t + 1} + \left( {\theta - 1} \right)\hat{r}_{t + 1}^{f} }}{{\theta^{2} \hat{\sigma }_{t + 1}^{2} }}$$

where $\gamma$ represents the risk aversion coefficient; $\theta$ is a leverage ratio that is set between 1 and 10, given the assumption that investors usually maintain a margin account at 10% level $\left( {\theta - 10} \right)$; $\hat{r}_{t + 1}$ is the realized volatility forecast at time $t + 1$; $\hat{r}_{t + 1}^{f}$ is a risk free asset (we used the US Treasury bill rate); and $\hat{\sigma }_{t + 1}^{2}$ is the estimate of the return volatility, which is estimated using a 30-day moving window of daily returns. The certainty equivalent return (CER) for investors’ optimal weight $\left( {w_{t} } \right)$ in Eq. (3) is defined as

4 $$CER = \overline{R}_{p} - 0.5\left( {{1 \mathord{\left/ {\vphantom {1 \gamma }} \right. \kern-0pt} \gamma }} \right)\sigma_{p}^{2}$$

where $\overline{R}_{p}$ and $\sigma_{p}^{2}$ are the out-of-sample period mean and variance, respectively, of the portfolio returns, $R_{p} = w\theta \left( {r - r^{f} } \right) + \left( {1 - w} \right)r^{f}$. The associated portfolio return variance is defined as $Var\left( {R_{p} } \right) = w^{2} \theta^{2} \sigma^{2}$, where $\sigma^{2}$ denotes the excess return volatility. The economic significance is consequently determined by maximizing the objective function of a utility as follows:

5 $$\begin{aligned} U\left( {R_{p} } \right) & = E\left( {R_{p} } \right) - 0.5\left( {{1 \mathord{\left/ {\vphantom {1 \gamma }} \right. \kern-0pt} \gamma }} \right)Var\left( {R_{p} } \right) \\ & = w\theta \left( {r - r^{f} } \right) + \left( {1 - w} \right)r^{f} - 0.5\left( {{1 \mathord{\left/ {\vphantom {1 \gamma }} \right. \kern-0pt} \gamma }} \right)w^{2} \theta^{2} \sigma^{2} \\ \end{aligned}$$

We report the portfolio returns, the associated volatility, as well as the certainty equivalent returns and the Sharpe ratio (SR), which is computed as $SR = {{\left( {R_{p} - r^{f} } \right)} \mathord{\left/ {\vphantom {{\left( {R_{p} - r^{f} } \right)} {\sqrt {Var\left( {R_{p} } \right)} }}} \right. \kern-0pt} {\sqrt {Var\left( {R_{p} } \right)} }}$. We evaluate economic gains based on the model construct with the maximum returns, CER and SR, and the lowest volatility (see Liu et al. 2019). Table 3 shows the economic significance of incorporating Bitcoin price as a predictor in our WN-Type distributed lag model framework for predicting realized volatility of stocks in US sectors (Composite, Consumer Discretionary, Consumer Staples, Energy, Financials, Health Care, Industrials, Information Technology, Materials, Real Estate, Telecommunication Services, and Utilities) when the leverage parameter is set to 6 and 8.

**Table 3:** Economic significance

Sector stock	Model	Returns	Volatility	CER	SR	Returns	Volatility	CER	SR
		$\varvec{Gamma} = {\mathbf{3}}\;\varvec{and}\;\varvec{Theta} = {\mathbf{6}}$				$\varvec{Gamma} = {\mathbf{3}}\;\varvec{and}\;\varvec{Theta} = {\mathbf{8}}$			
Composite	HA	0.1582	6.3683	0.1569	0.0518	0.2180	11.0590	0.2166	0.0573
	WN	0.3786	9.9721	0.3772	**0.1112**	0.4942	18.0405	0.4928	**0.1099**
Consumer Discretionary	HA	0.4717	78.2085	0.4686	0.0502	0.6115	139.5238	0.6085	0.0494
	WN	− 2.4630	121.1427	− 2.4670	− 0.2263	− 3.3025	215.4363	− 3.3065	− 0.2269
Consumer Staples	HA	− 0.3913	10.6121	− 0.3919	− 0.1286	− 0.5171	18.6920	− 0.5177	− 0.1260
	WN	0.2351	3.2580	0.2344	**0.1150**	0.3054	5.7784	0.3047	**0.1156**
Energy	HA	0.4169	50.9515	0.4145	0.0545	0.5448	90.7675	0.5424	0.0543
	WN	− 3.8934	69.5225	− 3.8960	− 0.4702	− 5.1918	123.5344	− 5.1944	− 0.4696
Financials	HA	− 0.3992	12.8728	− 0.4004	− 0.1189	− 0.5129	22.0292	− 0.5140	− 0.1151
	WN	− 0.5381	37.2313	− 0.5394	− **0.0927**	− 0.7365	66.3159	− 0.7378	− **0.0938**
Health Care	HA	− 1.1088	29.4945	− 1.1097	− 0.2092	− 1.4770	52.4270	− 1.4778	− 0.2078
	WN	− 0.6532	29.7704	− 0.6546	− **0.1248**	− 0.8836	52.8847	− 0.8851	− **0.1253**
Industrials	HA	− 0.7841	20.0793	− 0.7856	− 0.1811	− 1.0216	34.3436	− 1.0230	− 0.1790
	WN	− 0.1195	33.2669	− 0.1214	− **0.0255**	− 0.1797	59.6844	− 0.1816	− **0.0268**
Information Technology	HA	0.5007	12.2098	0.4972	0.1354	0.6604	21.5676	0.6569	0.1363
	WN	− 0.1611	43.8404	− 0.1648	− 0.0285	− 0.2399	79.7341	− 0.2436	− 0.0299
Materials	HA	− 0.2289	19.4588	− 0.2300	− 0.0581	− 0.3026	34.2687	− 0.3038	− 0.0564
	WN	− 0.0533	18.5352	− 0.0543	− **0.0188**	− 0.0884	33.1728	− 0.0895	− **0.0201**
Real Estate	HA	− 1.3669	29.4363	− 1.3680	− 0.2570	− 1.8207	52.2294	− 1.8218	− 0.2557
	WN	− 0.9325	25.9896	− 0.9334	− **0.1883**	− 1.2618	46.2730	− 1.2627	− **0.1895**
Telecommunication Services	HA	0.3155	73.2586	0.3129	0.0336	0.4156	130.2285	0.4130	0.0340
	WN	− 0.8051	74.4048	− 0.8076	− 0.0965	− 1.0965	133.5648	− 1.0990	− 0.0973
Utilities	HA	0.2550	6.5103	0.2536	0.0891	0.3363	11.6497	0.3349	0.0905
	WN	0.4724	10.3612	0.4710	**0.1382**	0.6230	18.6182	0.6216	**0.1380**

HA is the historical average model while WN is the Westerlund and Narayan (2012, 2015) type distributed lag model that accommodates salient data features such as endogeneity, persistence, conditional heteroscedasticity and structural breaks. A given predictive model that incorporates Bitcoin (logged) as a predictor is said to yield economic gains over the compared benchmark whenever such model construct yields maximum returns, CER and SR; and minimum volatility. The figures in bold letterings are cases where our WN-Type predictive model provides some economic gains over the benchmark historical average model. Also, the cases of negative SR indicate that the returns of the corresponding stocks are lower than the risk free asset used in the computation of economic significance; however, the decision remains based on the maximum SR

Table 3 shows that when the leverage parameter is set to 6, our WN-Type distributed lag model that incorporates Bitcoin price as a predictor variable provides higher economic gains but with higher risks (except in the cases of Consumer Staples and Real Estate) than the benchmark historical average model in all cases except Consumer Discretionary, Energy, Information Technology, and Telecommunications services. We also see negative Sharpe Ratios in some cases (Consumer Discretionary, Consumer Staples, Energy, Financials, Health Care, Industrials, Information Technology, Materials, Real Estate, and Telecommunication Services), indicating that US sector stock returns are generally lower than the risk free asset. In other words, while the direction of Bitcoin’s impact on various US sectors is consistent for the in-sample case, the out-of-sample utility gains differ in terms of how they compare to the benchmark model that excludes crypto risk. From an investment standpoint, the latter is more insightful because profit-maximizing investors should pay more attention to sectors that clearly offer higher out-of-sample excess returns after accounting for crypto risk, regardless of in-sample estimates. When the leverage parameter is set to 8, the stance of economic gains does not change, as we see the same feats across all US sector stocks. According to the preceding, incorporating the Bitcoin price provides some economic gains regardless of the set leverage parameter, with higher gains being mostly associated with higher risks. In conclusion, our predictive model outperforms the benchmark historical average model both statistically and economically across in-sample and out-of-sample forecast horizons.

### Additional results

Two robustness analyses are performed. In the first step, we re-estimate the US sectoral stock realized volatility–Bitcoin nexus using a model-based volatility measure derived from the GARCH(1,1) model’s conditional variance. In the second, we use an 80:20 data split instead of the 75:25 data split used in the main analysis. We report the in-sample predictability and the forecast performances using the relative RMSE and the CW (2007) statistic for pairwise model comparison, as we did in the main estimation.

Tables 4 (for the case of a 75:20 data split with GARCH-based volatility measure) and 5 (for the case of an 80:20 data split with 20-days realized volatility) show the estimated results. In both cases, we find that evidence of in-sample predictability persists regardless of the volatility proxy and data splits used. This evidence of in-sample predictability of Bitcoin prices for various proxies of US stock realized volatility extends beyond the in-sample to the out-of-sample, as evidenced by our WN-Type distributed lag model’s consistent outperformance over the historical average across stock sector indices and the aggregate market index. Implicitly, our predictability and forecast performance results are not affected by the choice of realized volatility measures and data splits, indicating their robustness. However, our results on economic gains are somewhat sensitive to the choice of volatility measures (see Table 6) but robust to the data split (see Table 7). The economic gains of the WN-Type model are retained regardless of sample split choice. However, for the model-based volatility measure, our proposed model’s ability to provide higher economic gains than the benchmark model decreases when compared to the model-free approach involving the RV measure. This implies that when modeling stock volatility, care must be taken because the method of volatility generation appears to affect economic gains.

## Conclusion

This study examines the relationship between realized volatility of US stock returns across sectors and Bitcoin prices. Using the WN-Type predictive model, this is an attempt to determine the predictive potential of Bitcoin prices for the realized volatility of US stock returns while controlling for potential biases arising from model mis-specification and/or variable omission. The analyses are carried out for both in-sample and out-of-sample periods and multiple forecast horizons. The relative RMSE and the pairwise CW (2007) test statistics are used to evaluate forecast performance. Furthermore, the potential utility gains of monitoring Bitcoin prices when making investment decisions in the US stock market are considered. At least three significant structural breaks in the regression involving realized volatility and Bitcoin prices, one of which corresponds to the period following the WHO’s announcement of the COVID-19 pandemic. Consequently, incorporating these observed breaks into the model framework, which already accounts for other salient features such as endogeneity, persistence, and conditional heteroscedasticity, is hypothesized to improve predictability outcomes.

Our results are summarized below. First, Bitcoin prices have an inverse relationship with the realized volatility of US stocks. This indicates that falling Bitcoin prices may increase volatility in the US sector stock market due to improved trading in the latter. Second, in terms of out-of-sample forecast performance, the analysis shows that our predictive model that accounts for Bitcoin prices and other observed salient data features consistently outperforms the benchmark historical average model that does not consider these data. This conclusion holds true across multiple sectors of the US stock market and forecast horizons. Third, in terms of economic significance, incorporating Bitcoin prices as a predictor yields higher economic gains in a larger proportion of US sector stocks under alternative leverage ratio assumptions. Based on the foregoing, we can conclude that using Bitcoin prices to forecast the realized volatility of US stocks will not only improve forecasts but will also result in higher economic gains. Accordingly, investors and portfolio managers seeking to maximize returns in the US stock market should pay attention to the price dynamics in the Bitcoin market, as they have the potential to significantly influence the volatility formation of US stocks. This assists market participants in fine-tuning their forecasting models, which has implications for risk management, financial derivatives, and market efficiency. Similarly, practitioners and academics who are constantly involved in financial market analyses may find our proposed model and the various conclusions interesting, especially in terms of producing more accurate forecasts when analyzing the riskiness of the US stock market. This is important given the recent view and evidences that cryptocurrencies in general and Bitcoin in particular are becoming mainstream and thus closer to the global financial system, including the US stock market. Accordingly, these digital assets should be monitored carefully by central banks and regulatory bodies as they can pose risk to the financial system and thus to financial stability, which also requires a tighter regulation of cryptocurrencies in the same way as stock markets, without ignoring some of their particular features.

Future research should consider whether the above findings can be generalized to other stock markets in Europe and Asia. In addition, it could be interesting to assess the response of emerging markets to the dynamics of the Bitcoin market, while also investigating whether markets’ responses are symmetrical or asymmetrical, given the bullish and bearish nature of financial markets.

## Data Availability

Data used in the study are secondary published data extracted from DataStream. However, they are available on request from the authors. The models or methodology used in the study are not registered.

## Appendix A: Additional information on the WN-type distributed lag model

Let us construct a simple predictive model for the connection between Bitcoin and the stock market volatility as given below:

A1 $$RV_{t} = \omega + \beta btc_{t - 1} + \mu_{t} ;\quad \varepsilon_{t} \sim N\left( {0,\sigma_{\varepsilon }^{2} } \right)$$

where $RV_{t}$ is the realized volatility of stock returns and $btc_{t}$ is the Bitcoin prices (expressed in natural logarithm). We note from our preliminary analyses that $btc_{t}$ exhibits some persistence effect which is based on the test equation in (A2):

A2 $$btc_{t} = \phi + \rho btc_{t - 1} + \nu_{t} ;\quad \nu_{t} \sim N\left( {0,\sigma_{\nu }^{2} } \right)$$

The presence of persistence effect in $btc_{t}$ introduces some endogeneity bias into the model in which $\mu_{t}$ and $\nu_{t}$ are correlated. Therefore, Westerlund and Narayan (2012, 2015) suggest formulating (A3) in order to accommodate this inherent correlation between the two disturbances as well as the associated endogeneity bias:

A3 $$\mu_{t} = \gamma \nu_{t} + \varepsilon_{t}$$

Based on (A1), $\mu_{t}$ can be written as $\mu_{t} = RV_{t} - \omega - \beta \,btc_{t - 1}$ and $\nu_{t}$ in (A2) as $\nu_{t} = btc_{t} - \phi - \rho btc_{t - 1}$. By introducing these respective representations for $\mu_{t}$ and $\nu_{t}$ into Eq. (A3) and simplifying further, we have:

A4 $$RV_{t} = \alpha + \beta \,btc_{t - 1} + \gamma \left( {btc_{t} - \rho btc_{t - 1} } \right) + \varepsilon_{t}$$

where $\alpha = \omega - \phi \gamma$. Note that (A4) is not different from Eq. (1) in the main text except that we further incorporate the first lag of $RV_{t}$ to account for additional persistence effect and structural breaks to deal with important structural shifts in the model.

## Appendix B

See Tables 4, 5, 6 and 7.

**Table 4:** Predictability and forecast evaluation (75:25 data split with GARCH based realized volatility)

Sectors	PANEL A	PANEL B Relative RMSE				PANEL C Clark and West (2007)			
	Parameter estimate								
		In sample	Out-of-sample			In sample	Out-of-sample		
			$\varvec{h} = {\mathbf{30}}$	$\varvec{h} = {\mathbf{60}}$	$\varvec{h} = {\mathbf{120}}$		$\varvec{h} = {\mathbf{30}}$	$\varvec{h} = {\mathbf{60}}$	$\varvec{h} = {\mathbf{120}}$
Composite	− 3.36E−05*** [3.51E−07]	9.67E−01	9.66E−01	9.65E−01	9.63E−01	3.71E−08*** [2.72E−09]	3.65E−08*** [2.63E−09]	3.57E−08*** [2.54E−09]	3.50E−08*** [2.38E−09]
Consumer discretionary	− 4.86E−05*** [8.47E−07]	9.79E−01	9.78E−01	9.79E−01	9.78E−01	2.19E−08*** [9.74E−10]	2.17E−08*** [9.41E−10]	2.09E−08*** [9.30E−10]	2.07E−08*** [8.77E−10]
Consumer staples	− 2.97E−05*** [4.33E−07]	9.65E−01	9.80E−01	1.02E+00	1.08E+00	1.34E−08*** [2.20E−09]	1.46E−08*** [2.14E−09]	1.57E−08*** [2.08E−09]	1.84E−08*** [1.98E−09]
Energy	− 1.41E−04*** [2.21E−07]	9.27E−01	9.30E−01	9.33E−01	9.41E−01	3.68E−07*** [1.48E−08]	3.51E−07*** [1.46E−08]	3.37E−07*** [1.43E−08]	3.05E−07*** [1.39E−08]
Financials	− 1.41E−04*** [3.71E−06]	1.01E+00	1.01E+00	1.01E+00	1.01E+00	2.21E−08*** [7.10E−09]	2.35E−08*** [6.85E−09]	2.45E−08*** [6.62E−09]	2.87E−08*** [6.21E−09]
Health Care	− 1.07E−04*** [9.80E−07]	9.79E−01	9.78E−01	9.77E−01	9.73E−01	9.63E−09*** [3.92E−10]	9.69E−09*** [3.79E−10]	9.72E−09*** [3.66E−10]	1.00E−08*** [3.45E−10]
Industrials	− 1.22E−05*** [5.91E−07]	9.97E−01	9.95E−01	9.95E−01	9.93E−01	1.81E−08*** [1.93E−09]	1.92E−08*** [1.87E−09]	2.00E−08*** [1.82E−09]	2.26E−08*** [1.74E−09]
Info. Technology	2.60E−06*** [1.40E−07]	9.97E−01	9.96E−01	9.96E−01	9.96E−01	4.03E−08*** [2.54E−09]	3.92E−08*** [2.46E−09]	3.79E−08*** [2.38E−09]	3.60E−08*** [2.24E−09]
Materials	− 4.26E−05*** [4.09E−07]	8.69E−01	8.71E−01	8.78E−01	8.89E−01	1.16E−07*** [1.85E−08]	1.13E−07*** [1.79E−08]	1.11E−07*** [1.72E−08]	1.09E−07*** [1.61E−08]
Real Estate	9.67E−06*** [1.86E−07]	1.01E+00	1.01E+00	1.01E+00	1.01E+00	8.24E−08*** [1.31E−08]	8.13E−08*** [1.26E−08]	8.23E−08*** [1.22E−08]	1.38E−08*** [3.46E−09]
Telecommunications	− 5.80E−05*** [8.16E−07]	9.86E−01	9.87E−01	9.87E−01	9.87E−01	1.08E−08*** [5.67E−10]	1.04E−08*** [5.52E−10]	9.98E−09*** [5.37E−10]	9.28E−09*** [5.09E−10]
Utilities	− 4.27E−05*** [1.21E−08]	9.67E−01	9.68E−01	9.72E−01	9.77E−01	1.72E−08*** [9.53E−10]	3.23E−08*** [2.94E−09]	3.31E−08*** [2.84E−09]	3.58E−08*** [2.68E−09]

The results presented on the table are from the estimation of the WN-Type distributed lag predictive model for Bitcoin prices using realized volatilities of US sectoral stock returns singly as predictors, while simultaneously accounting for inherent persistence, endogeneity, conditional heteroscedasticity and structural breaks. The table comprises three panels: PANEL A presents the in-sample predictability of the realized volatility of US sectoral stock returns for log-transformed Bitcoin price; PANEL B presents the relative root mean square error that compares our WN-Type distributed lag model with the historical average model; while PANEL C presents the Clark and West (2007) test statistics that entails a pairwise comparison of our predictive model with the benchmark historical average model. Under Panels A and C, each cell contain the estimates and the corresponding standard errors in square brackets; while the *** indicates statistical significance at 1% level. Under the PANEL B, values less than unity indicate preference of our predictive model over the benchmark Historical average model; while under PANEL C, our predictive model is adjudged the preferred when the CW statistic is positive and statistically significant

**Table 5:** Predictability and forecast evaluation (80:20 data split with 20-day annualized realized volatility)

Sectors	PANEL A	PANEL B Relative RMSE				PANEL C Clark and West (2007)			
	Parameter estimate								
		In sample	Out-of-sample			In sample	Out-of-sample		
			$\varvec{h} = {\mathbf{30}}$	$\varvec{h} = {\mathbf{60}}$	$\varvec{h} = {\mathbf{120}}$		$\varvec{h} = {\mathbf{30}}$	$\varvec{h} = {\mathbf{60}}$	$\varvec{h} = {\mathbf{120}}$
Composite	− 0.2168*** [0.0037]	0.9086	0.9085	0.9075	0.9072	0.1722*** [0.0114]	0.1670*** [0.0111]	0.1628*** [0.0107]	0.1542*** [0.0101]
Consumer discretionary	− 0.2021*** [0.0004]	0.8633	0.8664	0.8660	0.8724	0.1803*** [0.0114]	0.1733*** [0.0111]	0.1682*** [0.0108]	0.1560*** [0.0102]
Consumer staples	− 0.1787*** [0.0004]	0.9178	0.9515	0.9685	0.9752	0.1069*** [0.0086]	0.1068*** [0.0083]	0.1105*** [0.0081]	0.1198*** [0.0077]
Energy	− 0.6393*** [0.0076]	0.6823	0.6826	0.6856	0.6978	1.0813*** [0.0866]	1.0460*** [0.0839]	1.0110*** [0.0814]	0.9468*** [0.0768]
Financials	− 0.3433*** [0.0029]	0.7630	0.7627	0.7627	0.7649	0.4771*** [0.0447]	0.4624*** [0.0432]	0.4486*** [0.0419]	0.4208*** [0.0395]
Health Care	− 0.3129*** [0.0027]	0.9202	0.9208	0.9165	0.9084	0.0770*** [0.0056]	0.0797*** [0.0054]	0.0835*** [0.0053]	0.0864*** [0.0050]
Industrials	− 0.3928*** [0.0019]	0.7608	0.7612	0.7591	0.7600	0.3900*** [0.0398]	0.3794*** [0.0385]	0.3712*** [0.0373]	0.3506*** [0.0351]
Info. Technology	− 0.2999*** [0.0030]	0.9158	0.9199	0.9188	0.9161	0.2090*** [0.0136]	0.1999*** [0.0133]	0.1952*** [0.0128]	0.1880*** [0.0121]
Materials	− 0.2377*** [0.0021]	0.8500	0.8499	0.8493	0.8496	0.2436*** [0.0156]	0.2360*** [0.0151]	0.2292*** [0.0147]	0.2161*** [0.0139]
Real Estate	− 0.2423*** [0.0005]	0.7951	0.7952	0.7958	0.7964	0.3844*** [0.0339]	0.3716*** [0.0328]	0.3599*** [0.0318]	0.3392*** [0.0300]
Telecommunications	− 0.2381*** [0.0011]	0.8657	0.8742	0.8753	0.8644	0.1217*** [0.0104]	0.1204*** [0.0100]	0.1210*** [0.0097]	0.1254*** [0.0092]
Utilities	− 0.2168*** [0.0037]	0.8456	0.8455	0.8451	0.8450	0.1722*** [0.0114]	0.1670*** [0.0111]	0.1628*** [0.0107]	0.1542*** [0.0101]

The results presented on the table are from the estimation of the WN-Type distributed lag predictive model for Bitcoin prices using realized volatilities of US sectoral stock returns singly as predictors, while simultaneously accounting for inherent persistence, endogeneity, conditional heteroscedasticity and structural breaks. The table comprises three panels: PANEL A presents the in-sample predictability of the realized volatility of US sectoral stock returns for log-transformed Bitcoin price; PANEL B presents the relative root mean square error that compares our WN-Type distributed lag model with the historical average model; while PANEL C presents the Clark and West (2007) test statistics that entails a pairwise comparison of our predictive model with the benchmark historical average model. Under Panels A and C, each cell contain the estimates and the corresponding standard errors in square brackets; while the *** indicates statistical significance at 1% level. Under the PANEL B, values less than unity indicate preference of our predictive model over the benchmark Historical average model; while under PANEL C, our predictive model is adjudged the preferred when the CW statistic is positive and statistically significant

**Table 6:** Economic significance (75:25 data split with GARCH based realized volatility)

Sector stock	Model	Returns	Volatility	CER	SR	Returns	Volatility	CER	SR
		$\varvec{Gamma} = {\mathbf{3}}\;\varvec{and}\;\varvec{Theta} = {\mathbf{6}}$				$\varvec{Gamma} = {\mathbf{3}}\;\varvec{and}\;\varvec{Theta} = {\mathbf{8}}$			
Composite	HA	0.2527	1.89E−05	0.2527	51.8445	0.3171	3.35E−05	0.3171	50.0171
	WN	0.2537	1.85E−05	0.2537	**52.6391**	0.3185	3.28E−05	0.3185	**50.7792**
Consumer discretionary	HA	0.2299	2.88E−05	0.2299	37.7162	0.2879	5.12E−05	0.2879	36.3875
	WN	0.2346	2.94E−05	0.2346	**38.2143**	0.2938	5.22E−05	0.2938	**36.8585**
Consumer staples	HA	0.1150	9.44E−07	0.1150	90.0166	0.1401	1.68E−06	0.1401	86.8932
	WN	0.1619	4.24E−06	0.1619	65.2402	0.2002	7.54E−06	0.2002	62.9133
Energy	HA	0.1053	1.73E−05	0.1053	18.6993	0.1276	3.07E−05	0.1276	18.0533
	WN	0.1020	1.66E−05	0.1020	18.2954	0.1234	2.94E−05	0.1234	17.6695
Financials	HA	0.1856	1.55E−05	0.1856	40.1856	0.2309	2.75E−05	0.2309	38.7731
	WN	0.1913	1.61E−05	0.1913	**40.8373**	0.2382	2.86E−05	0.2382	**39.3970**
Health Care	HA	0.2108	4.06E−06	0.2108	90.9209	0.2633	7.23E−06	0.2633	87.7234
	WN	0.2061	4.06E−06	0.2061	88.6021	0.2573	7.22E−06	0.2573	85.4844
Industrials	HA	0.2554	1.08E−05	0.2554	69.3924	0.3206	1.92E−05	0.3206	66.9491
	WN	0.2635	1.10E−05	0.2635	**71.1749**	0.3309	1.95E−05	0.3309	**68.6510**
Information Technology	HA	0.0907	4.49E−05	0.0907	9.4319	0.1089	7.98E−05	0.1089	9.1118
	WN	0.0897	4.49E−05	0.0897	9.2772	0.1076	7.98E−05	0.1076	8.9623
Materials	HA	0.1730	1.15E−05	0.1730	42.8836	0.2147	2.05E−05	0.2147	41.3808
	WN	0.2146	1.97E−05	0.2146	42.1344	0.2681	3.51E−05	0.2681	40.6387
Real Estate	HA	0.1176	1.39E−06	0.1176	76.5535	0.1435	2.46E−06	0.1435	73.9247
	WN	0.1542	6.10E−06	0.1542	51.2772	0.1905	1.09E−05	0.1905	49.4629
Telecommunication Services	HA	0.1960	1.10E−05	0.1960	50.8572	0.2443	1.95E−05	0.2443	49.0645
	WN	0.1958	1.11E−05	0.1958	50.5779	0.2440	1.97E−05	0.2440	48.7952
Utilities	HA	0.2332	4.11E−06	0.2332	101.4970	0.2921	7.30E−06	0.2921	97.9237
	WN	0.2456	4.63E−06	0.2456	101.3130	0.3080	8.24E−06	0.3080	97.7250

HA is the historical average model while WN is the Westerlund and Narayan (2012, 2015) type distributed lag model that accommodates salient data features such as endogeneity, persistence, conditional heteroscedasticity and structural breaks. A given predictive model that incorporates Bitcoin (logged) as a predictor is said to yield economic gains over the compared benchmark whenever such model construct yields maximum returns, CER and SR; and minimum volatility. The figures in bold letterings are cases where our WN-type predictive model provides some economic gains over the benchmark historical average model. Also, the cases of negative SR indicate that the returns of the corresponding stocks are lower than the risk free asset used in the computation of economic significance; however, the decision remains based on the maximum SR

**Table 7:** Economic significance (80:20 Data Split with 20-day annualized realized volatility)

Sector stock	Model	Returns	Volatility	CER	SR	Returns	Volatility	CER	SR
		$\varvec{Gamma} = {\mathbf{3}}\;\varvec{and}\;\varvec{Theta} = {\mathbf{6}}$				$\varvec{Gamma} = {\mathbf{3}}\;\varvec{and}\;\varvec{Theta} = {\mathbf{8}}$			
Composite	HA	− 1.6221	3.97E+01	− 1.6233	− 0.2633	− 2.1671	7.06E+01	− 2.1683	− 0.2624
	WN	− 0.9601	3.00E+01	− 0.9612	− **0.1820**	− 1.2704	5.26E+01	− 1.2716	− **0.1803**
Consumer Discretionary	HA	− 1.3803	8.61E+01	− 1.3831	− 0.1528	− 1.8357	1.52E+02	− 1.8386	− 0.1517
	WN	− 1.2221	8.67E+01	− 1.2251	− **0.1353**	− 1.6256	1.53E+02	− 1.6286	− **0.1342**
Consumer Staples	HA	− 0.6852	1.11E+01	− 0.6859	− 0.2164	− 0.9199	1.98E+01	− 0.9206	− 0.2154
	WN	− 1.9433	3.00E+01	− 1.9442	− 0.3616	− 2.6162	5.33E+01	− 2.6172	− 0.3635
Energy	HA	0.1294	7.66E+00	0.1266	0.0333	0.1830	1.26E+01	0.1802	0.0410
	WN	− 1.1441	5.24E+01	− 1.1470	− 0.1632	− 1.5271	9.29E+01	− 1.5299	− 0.1623
Financials	HA	− 0.2931	4.07E+01	− 0.2948	− 0.0518	− 0.3833	7.11E+01	− 0.3850	− 0.0499
	WN	− 0.4288	3.23E+01	− 0.4305	− 0.0821	− 0.5669	5.65E+01	− 0.5686	− 0.0804
Health Care	HA	− 1.1077	8.78E+00	− 1.1081	− 0.3865	− 1.4868	1.57E+01	− 1.4872	− 0.3852
	WN	0.3674	3.43E+00	0.3666	**0.1781**	0.4776	6.10E+00	0.4768	**0.1782**
Industrials	HA	− 2.0448	5.71E+01	− 2.0467	− 0.2755	− 2.7280	1.02E+02	− 2.7298	− 0.2744
	WN	− 0.5362	2.83E+01	− 0.5380	− **0.1078**	− 0.7163	5.02E+01	− 0.7181	− **0.1064**
Information Technology	HA	− 1.3994	1.08E+02	− 1.4015	− 0.1381	− 1.8720	1.92E+02	− 1.8740	− 0.1377
	WN	− 0.5430	8.78E+01	− 0.5451	− **0.0619**	− 0.7297	1.56E+02	− 0.7318	− **0.0615**
Materials	HA	− 1.1526	2.21E+01	− 1.1533	− 0.2530	− 1.5410	3.93E+01	− 1.5417	− 0.2518
	WN	0.4039	3.98E+00	0.4031	**0.1837**	0.5424	6.96E+00	0.5417	**0.1914**
Real Estate	HA	− 1.4401	2.09E+01	− 1.4407	− 0.3234	− 1.9296	3.71E+01	− 1.9302	− 0.3228
	WN	− 1.2866	2.17E+01	− 1.2873	− **0.2841**	− 1.7162	3.85E+01	− 1.7169	− **0.2825**
Telecommunication Services	HA	− 1.8706	4.44E+01	− 1.8721	− 0.2864	− 2.4926	7.87E+01	− 2.4941	− 0.2851
	WN	0.6459	1.97E+01	0.6441	**0.1370**	0.8455	3.58E+01	0.8437	**0.1351**
Utilities	HA	− 0.7391	3.44E+01	− 0.7405	− 0.1324	− 0.9799	6.07E+01	− 0.9813	− 0.1306
	WN	− 0.5224	2.62E+01	− 0.5236	− **0.1093**	− 0.6884	4.59E+01	− 0.6897	− **0.1071**

HA is the historical average model while WN is the Westerlund and Narayan (2012, 2015) type distributed lag model that accommodates salient data features such as endogeneity, persistence, conditional heteroscedasticity and structural breaks. A given predictive model that incorporates Bitcoin (logged) as a predictor is said to yield economic gains over the compared benchmark whenever such model construct yields maximum returns, CER and SR; and minimum volatility. The figures in bold letterings are cases where our WN-type predictive model provides some economic gains over the benchmark historical average model. Also, the cases of negative SR indicate that the returns of the corresponding stocks are lower than the risk free asset used in the computation of economic significance; however, the decision remains based on the maximum SR

## Footnotes

1 The strong connection between extreme positive returns and the cross-sectional pricing of cryptocurrencies (see, Ozdamar et al. 2021) further underscores the significance of the crypto market in exhibiting some of the features typical of the conventional financial markets and therefore the possible connection between the two markets is not unexpected.

2 Previous studies have applied strategies based on technical analysis. Dibeh and Harmanani (2012) propose the stochastic chartist–fundamentalist model of speculative asset dynamics in financial markets, whereas Kouatli and Yunis (2021) consider stock trading decisions based on technical indicators.

3 Some studies consider cross listing and its potential effect on market efficiency and information spillovers across stocks (see, Sita and Abdallah 2014; Abed and Abdallah 2019; Abdallah et al. 2021).

4 For banking and monetary policies in a changing financial environment, see Shahin and El-Achkar (2017). Furthermore, Abosedra et al. (2021) provide evidence on the presence of the so-called uncertainty of the pandemic phenomenon.

5 Realized Volatility is computed by using the sum of square returns over 20 days, hence 20-day realized volatility. The 20-day realized volatility is informed by the nature of the data which is covering 5-days a week. Accordingly, 20 days is the number of days within 4 weeks (approximately 1 month), and thus the 20-day realized volatility should serve as a good measure of realized volatility for investors and other market participants observing the market dynamics before making investment decision.

6 Fang et al. (2022) provide a comprehensive survey on cryptocurrency trading.

7 https://www.washingtonpost.com/business/2022/01/22/crypto-crash-bitcoin-fed/.

8 Some of the attractions to Bitcoin relative to other crypto currencies (such as Ethereum, Monero, Dash, Ripple, and Litecoin) is well-documented in Mensi et al. (2021).

9 https://www.bloomberg.com/news/articles/2021-12-03/bitcoin-s-correlation-with-stocks-grows-as-risk-appetite-drops.

10 On a linked front, Kwon (2020) shows that the tail behavior of Bitcoin is associated with that of the S&P 500 index (see Kwon 2020). Bouri et al. (2022) point to significant co-movements in the higher-order moments of the returns of Bitcoin and the S&P 500 index.

11 Further, there is evidence of the impact of the pandemic on the cryptocurrency market (Shahzad et al. 2021b).

12 For a systematic review of blockchain, see Xu et al. (2019).

13 See “Appendix A”.

14 For technical details on how the daily Realized Volatility is computed, see https://www.realvol.com/VolFormula.htm.
